# Application of connectivity index of cubic fuzzy graphs for identification of danger zones of tsunami threat

**DOI:** 10.1371/journal.pone.0297197

**Published:** 2024-01-30

**Authors:** Xiaolong Shi, Saeed Kosari, Saira Hameed, Abdul Ghafar Shah, Samee Ullah

**Affiliations:** 1 Institute of Computing Science and Technology, Guangzhou University, Guangzhou, China; 2 Department of Mathematics, University of the Punjab, Quaid-e-Azam Campus, Lahore, Pakistan; Rikkyo University, JAPAN

## Abstract

Fuzzy graphs are very important when we are trying to understand and study complex systems with uncertain and not exact information. Among different types of fuzzy graphs, cubic fuzzy graphs are special due to their ability to represent the membership degree of both vertices and edges using intervals and fuzzy numbers, respectively. To figure out how things are connected in cubic fuzzy graphs, we need to know about cubic *α*−strong, cubic *β*−strong and cubic *δ*−weak edges. These concepts better help in making decisions, solving problems and analyzing things like transportation, social networks and communication systems. The applicability of connectivity and comprehension of cubic fuzzy graphs have urged us to discuss connectivity in the domain of cubic fuzzy graphs. In this paper, the terms partial cubic *α*−strong and partial cubic *δ*−weak edges are introduced for cubic fuzzy graphs. The bounds and exact expression of connectivity index for several cubic fuzzy graphs are estimated. The average connectivity index for cubic fuzzy graphs is also defined and some results pertaining to these concepts are proved in this paper. The results demonstrate that removing some vertices or edges may cause a change in the value of connectivity index or average connectivity index, but the change will not necessarily be related to both values. This paper also defines the concepts of partial cubic connectivity enhancing node and partial cubic connectivity reducing node and some related results are proved. Furthermore, the concepts of cubic *α*−strong, cubic *β*− strong, cubic *δ*−weak edge, partial cubic *α*−strong and partial cubic *δ*−weak edges are utilized to identify areas most affected by a tsunami resulting from an earthquake. Finally, the research findings are compared with the existing methods to demonstrate their suitability and creativity.

## 1 Introduction

Graph theory is a vital field in various domains including mathematics, engineering, physics, social sciences, biology, computer science and linguistics, etc. The notion of a fuzzy graph arises from the idea that networks can sometimes be unclear or uncertain. This is an important field of research. Traditional graphs are limited when it comes to capturing the uncertain nature of network measurements, like strong connections, accomplished individuals and influential figures in social networks. Fuzzy graphs, on the other hand, provide a better representation of these less clear aspects. The existence of uncertainty in certain aspects of graph theory problems has led to the development of fuzzy theory. In 1965, Zadeh [1] introduced the concept of fuzzy set (FS) theory as an extension of the classical notion of a set which provided a mathematical approach for decision-making problems using fuzzy descriptions. Building on this idea, Rosenfeld [2], Yeh and Bang [3] introduced fuzzy graph (FG) theory in 1975, utilizing the concept of FS and graph theory. Fuzzy graphs (FGs) have found numerous applications in various fields including broadcast communications, artificial reasoning, data hypothesis, neural systems, etc. Nawaz and Akram [4] introduced information system for a Pythagorean fuzzy soft set. Nawaz and Akram [5] gave the idea of competition graph and economic competition graph in fuzzy soft theory. Rashmanlou et al. [6] discussed the types of isomoprhism for irregular bipolar fuzzy graphs. Akram et al. [7] introduced innovative idea of complex Pythagorean fuzzy threshold graphs. Zeng et al. [8] discussed the concept of maximal product on two strong-(SVNGS) and maximal product of connected-SVNG. Broumi et al. [9] introduced the concept of Fermatean neutrosophic graphs and presented some operations on Fermatean neutrosophic graphs. Broumi et al. [10] determined the shortest path using an ant colony optimization algorithm with single value triangular neutrosophic numbers as arc weights. FG theory is a broad and significant concept in today’s research landscape. The fundamental and crucial aspect within this field revolves around connectivity. Connectivity is a fundamental and critical concept in the field of fuzzy graph theory. It plays an important role in our life problems e.g., potential flow problems, network routing etc. Mathew and Sunitha [11, 12] analyzed the concepts of edge, vertex and cycle connectivity. Banerjee [13], Tong and Zheng [14] also provided several algorithms for determining the connectivity of a FG. Different connectivity measures including connectivity index, Wiener index, domination number, topological indices, etc. are discussed in [15–18]. Measures of connectivity in rough fuzzy network models were studied by Akram and Zafar [19]. Hameed et al. [20] presented a new model of complex fuzzy threshold graph. Binu et al. [21] studied the connectivity index (${CI}^{\infty }$) of FGs and its application in human trafficking. Akram et al. [22] discussed the connectivity indices of *m*-polar fuzzy network model. They utilized these indices in a product manufacturing problem. In 2009, Mathew and Sunitha proposed the concept of different types of arcs including *α*-strong, *β*-strong and *δ*-edges in FG [23]. After that, in 2011, Karunambigai et al. [24] introduced different types of arcs in intuitionistic fuzzy graphs (IFGs). In 2021, Akram et al. [25] presented the concept of strong edges for *m*-polar FGs. Rao et al. [26] introduced the concept of different types of arcs in intuitionistic fuzzy graph. The connectivity index (${CI}^{\infty }$) for IFGs was studied by Naeem et al. [27], in 2021. Interval-valued fuzzy set (IVFS) an extension (the concept of fuzzy set by allowing membership degree to be expressed as interval instead of single point) was introduced by Zadeh [28]. In 2011, Akram and Dudek [29] defined different fuzzy graph operation on IVFGs. In 2020, Talebi et al. [30] also introduced new concepts of interval-valued intuitionistic fuzzy graphs (IVIFG). Rashmanlou and Jun [31] discussed complete IVFG. Talebi et al. [32] discussed interval-valued intuitionistic fuzzy competition graph of an interval-valued intuitionistic fuzzy digraph. Self centered IVFGs were discussed in [33]. Broumi et al. [34] introduced the interval-valued fermatean neutrosophic set, which deals with partial ignorance in true, false or uncertain regions independently for multi-decision processe.

In 2012, the concept of cubic fuzzy sets (CFSs) which combines IVFS and FS to provide a more general way of handling uncertainty, was introduced by Jun et al. [35]. They also defined some basic properties and operations enabling the use of CFSs in decision making to solve problems involving uncertain data. Cubic fuzzy sets offer distinct advantages over other types of fuzzy sets, like interval-valued or general fuzzy sets. Their unique shape and parameters provide exceptional flexibility in modeling uncertainty. They enable a more precise representation of intricate relationships within a specific domain. By enhancing decision-making and reasoning abilities, cubic fuzzy sets become invaluable tools in various fields that rely on accurately modeling uncertainty. The notion of cubic fuzzy graphs (CFGs) (by applying the CFS on graph) was presented by Rashid et al. [36] and Muhiuddin et al. [37]. Muhiuddin et al. [38] worked on cubic Pythagorean fuzzy graphs (CPFGs) and introduced certain fundamental operations such as semi-strong product, lexicographical product and symmetric difference of two CPFGs. Cubic planar graphs were investigated bu Muhiuddin et al. [39]. They utilized it in road network problem. Krishna et al. [40] worked on properties of an edge in regular CFG. Senapati et al. [41] presented the idea of of cubic sets in UP-subalgebras and consider the UP-ideals of a UP-algebra and investigated the Relationships between cubic UP-subalgebras and the cubic UP-ideals of a UP-algebra. In 2022, Shi et al. [42] presented the concept of ${CI}^{\infty }$ in CFGs. In real-world situations, we can effectively use fuzzy graph ideas to describe some phenomena and interval-valued graph concepts work better for others. However, for more complex phenomena that can’t be adequately represented by either of these approaches alone, we can turn to a combination of both, which we call cubic fuzzy graphs. An example of where this combined modeling approach is useful is in understanding tsunami threat problem. When we need to make decisions that involve considering the past, present and future all at once, cubic fuzzy graphs are quite handy. They provide a valuable tool for visually representing information that spans multiple time dimensions, giving us a comprehensive view of the situation at hand. In comparative view of neutrosophic fuzzy graphs and cubic fuzzy graphs, neutrosophic fuzzy graphs and cubic fuzzy graphs are distinct extensions of fuzzy graph theory. Neutrosophic fuzzy graphs introduce the concept of neutrosophic sets, allowing for a more nuanced representation of uncertainty through the inclusion of truth-membership, indeterminacy-membership, and falsity-membership degrees. On the other hand, cubic fuzzy graphs extend traditional fuzzy graphs by incorporating three membership degrees (lower interval-valued fuzzy membership, upper interval-valued fuzzy membership and fuzzy membership) for each pair of vertices. While neutrosophic fuzzy graphs emphasize the trichotomy of truth, indeterminacy and falsity, cubic fuzzy graphs focus on the triple-membership structure, enabling a richer characterization of relationships in uncertain environments. Cubic fuzzy graphs are well-suited for scenarios where a higher level of granularity in membership assignment is needed to reflect the complexity of uncertain information. Both models contribute valuable tools for modeling uncertainty and the choice between them depends on the specific nature of uncertainty being addressed and the desired level of detail in the representation of relationships.

### 1.1 Motivation and contribution

Cubic fuzzy graphs have more advantageous representation as compared to interval-valued fuzzy graphs and fuzzy graphs because they depict the membership degree of vertices and edges in both interval and fuzzy number forms. This improved representation enables a deeper and more detailed comprehension of the connections and uncertainties present within the structure of the graph. The following features of strong and weak edges in CFG theory motivate us to present this paper:

- In practical situations, some problems can be solved by using either FG or IVFG concepts, while more complex problems may require a combination of both. CFGs provide a useful tool to tackle such problems. For example, traffic flow modeling and earthquake modeling problems can be addressed with the help of CFGs.
- Given the extensive applications of strong and weak edges in crisp and fuzzy graphs across various fields, it is worthwhile to investigate their relevance to CFGs as well.
- It is observed that the definitions of cubic *α*−strong and cubic *δ*−weak edges for cubic graphs [42] are very strict. It may happen that a connected network may not have any such edges. In this situation, the decision making can be difficult in these connectivity problems. To overcome this problem, a more general model for strong edges has to be defined.
- It is also observed that concept of ${CI}^{\infty }$ and average connectivity index (${ACI}^{\infty }$) are well-documented in the literature for crisp and fuzzy graphs, but their counterparts for CFGs are not widely known. These concepts are essential for conducting a thorough investigation of connectivity in CFGs.
- The study of strong, weak edges and connectivity index can be implemented in variety of decision-making problems.

Given the extensive importance and broad applications of cubic *α*−strong, cubic *β*−strong and cubic *δ*−weak edges within fuzzy networks, we have introduced the notion of partial cubic *α*−strong and partial cubic *δ*−weak edges for CFGs. These partial edges prove beneficial in addressing practical issues where the concept of cubic *α*−strong, cubic *β*−strong and cubic *δ*−weak edges may not be applicable. Specifically, these concepts come into play when the *IVF*− connectivity strictly exceeds or falls below the *IVF*−membership value of an edge, while the *F*− connectivity equates to the *F*−membership value of that edge and vice versa. In scenarios where we have information about the past, future and current conditions of a model or problem, we can represent the past condition as a lower interval-valued fuzzy membership, the future condition as an upper interval-valued fuzzy membership and the present condition as a fuzzy membership value. Our objective is to scrutinize the problem by deducing lower interval-valued fuzzy connectivity, upper interval-valued fuzzy connectivity and fuzzy connectivity. Furthermore, we aim to make new predictions based on this analysis. In these situations, the *IVF*− connectivity strictly exceeding or falling below the *IVF*−membership value of an edge occurs, while the *F*− connectivity aligns with the *F*−membership value of that edge and vice versa. To tackle this issue effectively, we can employ the concept of partial cubic *α*−strong and partial cubic *δ*−weak edges. Such problems frequently arise in the analysis of transportation networks, decision-making under uncertainty and optimization scenarios. Utilizing these partial cubic edges allows for a more accurate and detailed depiction of the connections between nodes or edges, enabling better modeling and evaluation of uncertain or imprecise relationships. It’s important to note that throughout this study, we specifically focused on simple connected CFGs. The primary contributions of this paper are outlined below.

- Given the significant importance and numerous applications of strong and weak edges in fuzzy networks, the objective of this research paper is to investigate the concept of strong and weak edges in CFG.
- To propose the concept of partial cubic *α*− strong and partial cubic *δ*− weak edges for CFG.
- To study the connectivity index in CFG and to establish their bounds or exact expression for several families of CFG, e.g., for complete CFG, a CFG with underlying crisp tree and cubic fuzzy cycle.
- To determine the effect on connectivity index of CFG on removal of an edge.
- To define average connectivity index, partial cubic connectivity enhancing node (PCCEN) and partial cubic connectivity reducing node (PCCRN) for CFG.
- To provide a more comprehensive understanding of the behavior of complex systems modeled by CFGs and to develop better strategies for addressing real-world problems such as earthquakes in certain areas by using cubic *α*− strong edges, cubic *β*−strong edges, cubic *δ*−weak edges, partial cubic *α*−strong edges and partial cubic *δ*−weak edges.
- To demonstrate the novelty of our model, we compare our results with existing models.

This research work is structured as follows: Section 2 comprises necessary definitions and results for the production of the concept. In Section 3, we examine the partially strong and weak edges. In Section 4, we introduce the concept of bounds for the ${CI}^{\infty }$ of CFGs and present related results. Section 5 covers the ${ACI}^{\infty }$ of CFGs along with relevant findings. In Section 6, we discuss various kinds of edges which can be helpful to examine the areas affected by tsunami due to an earthquake. Section 7 presents a comprehensive analysis of our research work. Finally, in Section 8, we conclude our investigations. Throughout the paper, we use the abbreviations given in the Table 1.

**Table 1:** Abbreviations.

Description	Abbreviation	Description	Abbreviation
Fuzzy sets	FSs	Fuzzy set	FS
Fuzzy Graphs	FGs	Fuzzy Graph	FG
Interval-valued fuzzy set	IVFS	Intuitionistic fuzzy graph	IFG
Interval-valued intuitionistic fuzzy graphs	IVIFG	Cubic fuzzy sets	CFSs
Cubic fuzzy set	CFS	Cubic fuzzy graph	CFG
Cubic Pythagorean fuzzy graphs	CPFGs	Connectivity index	${CI}^{\infty }$
Strength of path	S(P)	Strength of connectedness	${CONN}_{T}^{\infty }$
Average connectivity index	${ACI}^{\infty }$	Partial cubic connectivity reducing node	PCCRN
Partial cubic connectivity enhancing node	PCCEN	Partial cubic connectivity enhancing graph	PCCEG
Partial cubic connectivity reducing graph	PCCRG		

## 2 Preliminaries

**Definition 1** [35] A CFS *X* on a non-empty set *V* is described as

$$X=\{\left\langle \left[\sigma^{-}\left(t_{w}\right),\sigma^{+}\left(t_{w}\right)\right],\sigma^{F}\left(t_{w}\right)\;\;\right\rangle |t_{w}\in V\},$$

where [*σ*^−^(*t*_*w*_), *σ*^+^(*t*_*w*_)] is named as *IVF*-membership value and $\sigma^{F}\left(t_{w}\right)$ is named as *F*-membership value of *t*_*w*_. The CFS *X* is referred as internal CFS if $\sigma^{F}\left(t_{w}\right)\in \left[\sigma^{-}\left(t_{w}\right),\sigma^{+}\left(t_{w}\right)\right]$ for *t*_*w*_ ∈ *V*, otherwise it is called external CFS.

**Definition 2** [37] A CFG over the set *V* is a pair T=(A,B), where *A* is a CFS in *V* and *B* is a CFS in *V* × *V*, so that for all (*t*_*w*−1_, *t*_*w*_) ∈ *A*

$$\begin{array}{ccc} \mu^{-}\left(t_{w-1},t_{w}\right) & \leq & \wedge \{\sigma^{-}\left(t_{w-1}\right),\sigma^{-}\left(t_{w}\right)\},\\ \mu^{+}\left(t_{w-1},t_{w}\right) & \leq & \wedge \{\sigma^{+}\left(t_{w-1}\right),\sigma^{+}\left(t_{w}\right)\},\\ \mu^{F}\left(t_{w-1},t_{w}\right) & \leq & \wedge \{\sigma^{F}\left(t_{w-1}\right),\sigma^{F}\left(t_{w}\right)\}, \end{array}$$

A CFG T=(A,B) is said to be complete if

$$\begin{array}{ccc} \mu^{-}\left(t_{w-1},t_{w}\right) & = & \wedge \{\sigma^{-}\left(t_{w-1}\right),\sigma^{-}\left(t_{w}\right)\},\\ \mu^{+}\left(t_{w-1},t_{w}\right) & = & \wedge \{\sigma^{+}\left(t_{w-1}\right),\sigma^{+}\left(t_{w}\right)\},\\ \mu^{F}\left(t_{w-1},t_{w}\right) & = & \wedge \{\sigma^{F}\left(t_{w-1}\right),\sigma^{F}\left(t_{w}\right)\} \end{array}$$

for all *t*_*w*−1_, *t*_*w*_ ∈ *A*.

**Definition 3** [37] A cubic fuzzy path P of length *n* is a sequence of distinct vertices $t_{0},t_{1},t_{2},\dot{\ldots },t_{n}$ with *μ*^+^(*t*_*w*−1_, *t*_*w*_) > 0, *μ*^−^(*t*_*w*−1_, *t*_*w*_)>0 and $\mu^{F}\left(t_{w-1},t_{w}\right)>0$ for $w=1,2,3,\dot{\ldots },n$. A cubic fuzzy path P is called cycle if *t*_0_ = *t*_*n*_.

The strength of cubic fuzzy path $P=t_{1},t_{2},t_{3},\dot{\ldots },t_{n}$ is defined as

$$S\left(P\right)=\langle \left[L^{-}\left(P\right),L^{+}\left(P\right)\right],L^{F}\left(P\right)\rangle ,$$

where

$$L^{+}\left(P\right)=\wedge_{w=1}^{n}\mu^{+}\left(t_{w-1},t_{w}\right),L^{-}\left(P\right)=\wedge_{w=1}^{n}\mu^{-}\left(t_{w-1},t_{w}\right),$$

$$L^{F}\left(P\right)=\wedge_{w=1}^{n}\mu^{F}\left(t_{w-1},t_{w}\right).$$

The Strength of connectedness $\left({CONN}_{T}^{\infty }\right)$ among the vertices *t*_*w*−1_ and *t*_*w*_ is defined as:

$$\begin{array}{ccc} & & {CONN}_{T}^{\infty }\left(t_{w-1},t_{w}\right)\\ & = & \left\langle \left[{CONN}_{T}^{-}\left(t_{w-1},t_{w}\right),{CONN}_{T}^{+}\left(t_{w-1},t_{w}\right)\right],{CONN}_{T}^{F}\left(t_{w-1},t_{w}\right)\right\rangle \end{array}$$

where

$$\begin{array}{c} \begin{array}{c} {CONN}_{T}^{+}\left(t_{w-1},t_{w}\right)\\ =\vee \{L^{+}\left(P\right):P\;\text{is}\;a\;\text{path}\;\text{between}\;t_{w-1}\;\text{and}\;t_{w}\}, \end{array} \end{array}$$

$$\begin{array}{c} \begin{array}{c} {CONN}_{T}^{-}\left(t_{w-1},t_{w}\right)\\ =\vee \{L^{-}\left(P\right):P\;\text{is}\;a\;\text{path}\;\text{between}\;t_{w-1}\;\text{and}\;t_{w}\}, \end{array} \end{array}$$

$$\begin{array}{c} \begin{array}{c} {CONN}_{T}^{F}\left(t_{w-1},t_{w}\right)\\ =\vee \{L^{F}\left(P\right):P\;\text{is}\;a\;\text{path}\;\text{between}\;t_{w-1}\;\text{and}\;t_{w}\}. \end{array} \end{array}$$

The path P between *t*_*w*−1_ and *t*_*w*_ with

$$L^{+}\left(P\right)={CONN}_{T}^{+}\left(t_{w-1},t_{w}\right)$$

is referred as $L^{+}$-stronger path. Similarly $L^{-}$-stronger and $L^{F}$-stronger paths are defined. The $L^{+}$-stronger, $L^{-}$-stronger and $L^{F}$-stronger paths are denoted by $P^{+},P^{-}$ and $P^{F}$, respectively.

**Definition 4** [42] Let T=(A,B) be a CFG and (*t*_*w*−1_, *t*_*w*_) ∈ *B*.

1. If $\mu^{+}\left(t_{w-1}t_{w}\right)>{CONN}_{T-t_{w-1}t_{w}}^{+}\left(t_{w-1},t_{w}\right),$
  $$\mu^{-}\left(t_{w-1}t_{w}\right)>{CONN}_{T-t_{w-1}t_{w}}^{-}\left(t_{w-1},t_{w}\right),$$
  $$\mu^{F}\left(t_{w-1}t_{w}\right)>{CONN}_{T-t_{w-1}t_{w}}^{F}\left(t_{w-1},t_{w}\right),$$
  then *t*_*w*−1_*t*_*w*_ is called cubic *α*-strong.
2. If $\mu^{+}\left(t_{w-1}t_{w}\right)={CONN}_{T-t_{w-1}t_{w}}^{+}\left(t_{w-1},t_{w}\right)$,
  $$\mu^{-}\left(t_{w-1}t_{w}\right)={CONN}_{T-t_{w-1}t_{w}}^{-}\left(t_{w-1},t_{w}\right),$$
  $$\mu^{F}\left(t_{w-1}t_{w}\right)={CONN}_{T-t_{w-1}t_{w}}^{F}\left(t_{w-1},t_{w}\right),$$
  then *t*_*w*−1_*t*_*w*_ is called cubic *β*-strong.
3. If $\mu^{+}\left(t_{w-1}t_{w}\right)<{CONN}_{T-t_{w-1}t_{w}}^{+}\left(t_{w-1},t_{w}\right),$
  $$\mu^{-}\left(t_{w-1}t_{w}\right)<{CONN}_{T-t_{w-1}t_{w}}^{-}\left(t_{w-1},t_{w}\right)$$
  and
  $$\mu^{F}\left(t_{w-1}t_{w}\right)<{CONN}_{T-t_{w-1}t_{w}}^{F}\left(t_{w-1},t_{w}\right)$$
  then *t*_*w*−1_*t*_*w*_ is called cubic *δ*- weak edge.

**Definition 5** [40] The order of a CFG T=(A,B) is defined by

$$O\left(T\right)=\langle \left[\sum_{i\in V}\sigma^{-}\left(i\right),\sum_{i\in V}\sigma^{+}\left(i\right)\right],\sum_{i\in V}\sigma^{F}\left(i\right)\rangle ,$$

and size of CFG is defined by

$$S\left(T\right)=\langle \left[\sum_{ij\in E}\sigma^{-}\left(ij\right),\sum_{ij\in E}\sigma^{+}\left(ij\right)\right],\sum_{ij\in E}\sigma^{F}\left(ij\right)\rangle .$$

**Definition 6** [42] A CFG T is referred to be

- *α*−saturated if at each node of σ*, there are incident *n* ≥ 1 *α*− strong edges to it.
- *β*-saturated if at each node of σ*, there are incident *n* ≥ 1 *β* strong edges to it.
- Saturated if it is *α*− as well as *β*−saturated.
- Unsaturated if it is neither *α* nor *β* saturated.

**Definition 7** [42] The connectivity index $\left({CI}^{\infty }\right)$ of CFG T=(A,B) is defined as:

$$\begin{array}{c} {CI}^{\infty }\left(T\right)=\left\langle \left[{CI}^{-}\left(T\right),{CI}^{+}\left(T\right)\right],{CI}^{F}\left(T\right)\right\rangle , \end{array}$$

where

$$\begin{array}{ccc} {CI}^{+}\left(T\right) & = & \sum_{(s,t)\in A}\sigma^{+}\left(s\right)\sigma^{+}\left(t\right){CONN}_{T}^{+}\left(s,t\right),\\ {CI}^{-}\left(T\right) & = & \sum_{(s,t)\in A}\sigma^{-}\left(s\right)\sigma^{-}\left(t\right){CONN}_{T}^{-}\left(s,t\right),\\ {CI}^{F}\left(T\right) & = & \sum_{(s,t)\in A}\sigma^{F}\left(s\right)\sigma^{F}\left(t\right){CONN}_{T}^{F}\left(s,t\right). \end{array}$$

## 3 Partial cubic *α*− Strong and *δ*− Weak edges

**Fig 1:**
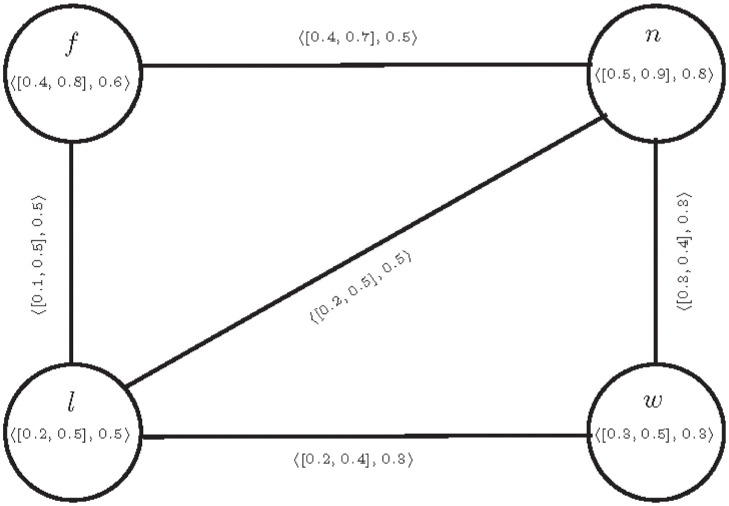
T=(E,S) .

The CF *α*− strong and CF *δ*− week edges are defined in [42]. But we note that there are CFGs which contain edges which are either *IVF*− *α*− strong and *F*−*β*− strong or *IVF*− *β*− strong and *F*− *α*− strong but not CF *α*− strong. These type of edges seem very close to CF *α*− strong edges and may be more useful in different CF connectivity problems. The following examples are helpful to understand this situation:

**Example 1** Consider a CFG T=(E,S) given in a Fig 1.

The connectivity for the pair *f*, *n* is computed as

$$\begin{array}{c} \begin{array}{c} \left[{CONN}_{T-(f,n)}^{-}\left(f,n\right),{CONN}_{T-(f,n)}^{+}\left(f,n\right)\right]=\left[0.1,0.5\right],{CONN}_{T-(f,n)}^{F}\left(f,n\right)=0.5,\\ {}[\mu^{-}\left(fn\right),\mu^{+}\left(fn\right)]=\left[0.4,0.7\right],\mu^{F}\left(fn\right)=0.5. \end{array} \end{array}$$

It is clear that the edge *fn* is *IVF*− *α*− strong edge but *F*− *β*− strong edge. We can see that If we slightly increase the value of *F*-membership of edge *fn*, then it becomes CF *α*− strong edge. So we can say that it is very close to CF *α*− strong edge.

**Fig 2:**
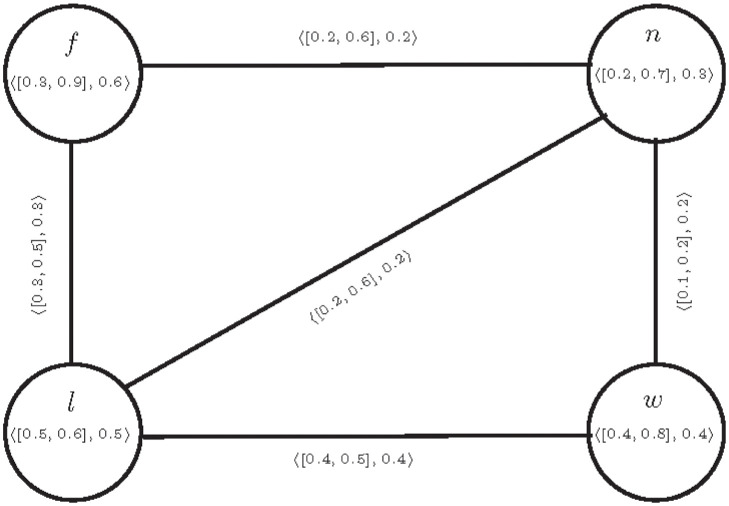
T=(E,S) .

**Example 2** Consider a CFG T=(E,S) given in a Fig 2.

The connectivity of pair *n*, *w* is calculated as

$$\begin{array}{c} \begin{array}{c} \left[{CONN}_{T-(n,w)}^{-}\left(n,w\right),{CONN}_{T-(n,w)}^{+}\left(n,w\right)\right]=\left[0.2,0.5\right],{CONN}_{T-(n,w)}^{F}\left(n,w\right)=0.2,\\ {}[\mu^{-}\left(nw\right),\mu^{+}\left(nw\right)]=\left[0.1,0.2\right],\mu^{F}\left(nw\right)=0.2. \end{array} \end{array}$$

It is clear that the edge *nw* is *IVF*− *δ*− weak and *F*− *β*− strong edge. Here if we slightly decrease the value of *F*-membership, then it becomes CF *δ*−weak edge. Above examples motivate to define the concept of partial cubic *α*− strong and partial cubic *δ*− weak edges.

**Definition 8** For a CF edge *t*_*w*−1_*t*_*w*_ in CFG, if one of the following holds, then *t*_*w*−1_*t*_*w*_ is called partial cubic *α*− strong edge.

1.
$\left[\mu_{T}^{-}\left(t_{w-1},t_{w}\right),\mu_{T}^{+}\left(t_{w-1},t_{w}\right)\right]\geq \left[{CONN}_{T-t_{w-1}t_{w}}^{-}\left(t_{w-1},t_{w}\right),{CONN}_{T-t_{w-1}t_{w}}^{+}\left(t_{w-1},t_{w}\right)\right]$ and

$\mu_{T}^{F}\left(t_{w-1}t_{w}\right)>{CONN}_{T-t_{w-1}t_{w}}^{F}\left(t_{w-1},t_{w}\right)$

2.
$\left[\mu_{T}^{-}\left(t_{w-1}t_{w}\right),\mu_{T}^{+}\left(t_{w-1}t_{w}\right)\right]>\left[{CONN}_{T-t_{w-1}t_{w}}^{-}\left(t_{w-1},t_{w}\right),{CONN}_{T-t_{w-1}t_{w}}^{+}\left(t_{w-1},t_{w}\right)\right]$ and

$\mu_{T}^{F}\left(t_{w-1}t_{w}\right)\geq {CONN}_{T-t_{w-1}t_{w}}^{F}\left(t_{w-1},t_{w}\right)$

In Example 1, the edge *fn* satisfies condition 2 of above definition, so the edge *fn* is partial cubic *α*− strong edge.

**Definition 9** For a CF edge *t*_*w*−1_*t*_*w*_ in CFG, if one of the following holds, then *t*_*w*−1_*t*_*w*_ is called partial cubic *δ*− weak edge.

1.
$\left[\mu_{T}^{-}\left(t_{w-1}t_{w}\right),\mu_{T}^{+}\left(t_{w-1}t_{w}\right)\right]\leq \left[{CONN}_{T-t_{w-1}t_{w}}^{-}\left(t_{w-1},t_{w}\right),{CONN}_{T-t_{w-1}t_{w}}^{+}\left(t_{w-1},t_{w}\right)\right]$ and

$\mu_{T}^{F}\left(t_{w-1}t_{w}\right)<{CONN}_{T-t_{w-1}t_{w}}^{F}\left(t_{w-1},t_{w}\right)$

2.
$\left[\mu_{T}^{-}\left(t_{w-1}t_{w}\right),\mu_{T}^{+}\left(t_{w-1}t_{w}\right)\right]<\left[{CONN}_{T-t_{w-1}t_{w}}^{-}\left(t_{w-1},t_{w}\right),{CONN}_{T-t_{w-1}t_{w}}^{+}\left(t_{w-1},t_{w}\right)\right]$ and

$\mu^{F}\left(t_{w-1}t_{w}\right)\leq {CONN}_{T-t_{w-1}t_{w}}^{F}\left(t_{w-1},t_{w}\right)$ .

In Example 2, the edge *nw* satisfies condition 2 of above definition, so the edge *nw* is a partial cubic *δ*− weak edge.

**Definition 10** A CFG T is referred to be

- Partial *α*−saturated if at each node of σ*, there are incident *n* ≥ 1 partial *α*− strong edges to it.
- *β*-saturated if at each node of σ*, there are incident *n* ≥ 1 *β* strong edges to it.
- Partial saturated if it is partial *α*−saturated as well as *β*−saturated.

## 4 Bounds for connectivity index of cubic fuzzy graph

In this section, we discuss bounds for the ${CI}^{\infty }$ of different families of CFGs.

**Theorem 1** Consider a complete CFG T=(σ,μ) with $d=\langle [d^{-},d^{+},d^{F}\rangle =\langle [min_{a\in \sigma^{*}}\sigma^{-}\left(v\right),min_{a\in \sigma^{*}}\sigma^{+}\left(v\right),min_{a\in \sigma^{*}}\sigma^{F}\left(v\right)\rangle$. Then

$$\begin{array}{c} \frac{n(n-1)}{2}d^{3}\leq {CI}^{\infty }\left(T\right)\leq O\left(T\right)\left(O\left(T\right)-d\right). \end{array}$$

**Proof** Let $\left\{t_{1}^{-},t_{2}^{-},\ldots ,t_{n}^{-}\right\}$, $\left\{t_{1}^{+},t_{2}^{+},\ldots ,t_{n}^{+}\right\}$, and $\left\{t_{1}^{F},t_{2}^{F},\ldots ,t_{n}^{F}\right\}$ be increasing sequences such that $t_{i}^{-}=\sigma^{-}\left(v_{i}\right)$, $t_{i}^{+}=\sigma^{+}\left(w_{i}\right)$ and $t_{i}^{F}=\sigma^{F}\left(u_{i}\right)$, respectively for *w*_*i*_, *v*_*i*_, *u*_*i*_ ∈ σ*. We note that for an edge *v*_1_*x*, ${CONN}^{+}\left(v_{1},x\right)=t_{1}^{-}$ and for an edge *v*_2_*y*, ${CONN}^{+}\left(v_{2},y\right)=t_{2}^{-}$ such that *y* ≠ *v*_1_. Similarly in this way, for edge *v*_*i*_*t*, ${CONN}^{+}\left(v_{i},t\right)=t_{i}^{-}$, where *t* ≠ *v*_*j*_, *j* < *i*. Thus we can write

$$\begin{array}{ccc} {CI}^{-}\left(T\right) & = & \sum_{i=1}^{n-1}\sum_{j=i+1}^{n}\sigma^{-}\left(v_{i}\right)\sigma^{-}\left(v_{j}\right){CONN}^{-}\left(v_{i},v_{j}\right)\\ & = & \sum_{i=1}^{n-1}{\left(t_{i}^{-}\right)}^{2}\sum_{j=i+1}^{n}t_{j}^{-}\\ & \geq & \sum_{i=1}^{n-1}{\left(t_{i}^{-}\right)}^{2}\sum_{j=i+1}^{n}t_{1}^{-}\geq \sum_{i=1}^{n-1}{\left(t_{i}^{-}\right)}^{2}t_{1}^{-}\left(n-i\right)\\ & \geq & {\left(d^{-}\right)}^{3}\sum_{i=1}^{n-1}\left(n-i\right)\geq \frac{{\left(d^{-}\right)}^{3}n\left(n-1\right)}{2}. \end{array}$$

Similarly, we have ${CI}^{-}\left(T\right)\geq \frac{{\left(d^{+}\right)}^{3}n\left(n-1\right)}{2}$ and ${CI}^{-}\left(T\right)\geq \frac{{\left(d^{F}\right)}^{3}n\left(n-1\right)}{2}$.

Now for upper bound,

$$\begin{array}{ccc} {CI}^{-}\left(T\right) & = & \sum_{i=1}^{n-1}\sum_{j=i+1}^{n}\sigma^{-}\left(v_{i}\right)\sigma^{-}\left(v_{j}\right){CONN}^{-}\left(v_{i},v_{j}\right)\\ & = & \sum_{i=1}^{n-1}{\left(t_{i}^{-}\right)}^{2}\sum_{j=i+1}^{n}t_{j}^{-}\leq \sum_{i=1}^{n-1}{\left(t_{i}^{-}\right)}^{2}\sum_{j=2}^{n}t_{j}^{-}\\ & \leq & \sum_{i=1}^{n-1}{\left(t_{i}^{-}\right)}^{2}\left(\sum_{j=1}^{n}t_{j}^{-}-t_{1}^{-}\right)\leq \sum_{i=1}^{n-1}\left(t_{i}^{-}\right)\left(O^{-}\left(T\right)-d^{-}\right)\\ & \leq & O^{-}\left(T\right)\left(O^{-}\left(T\right)-d^{-}\right). \end{array}$$

Similarly, we have ${CI}^{+}\left(T\right)\leq O^{+}\left(T\right)\left(O^{+}\left(T\right)-d^{+}\right)$ and ${CI}^{F}\left(T\right)\leq O^{F}\left(T\right)\left(O^{F}\left(T\right)-d^{F}\right).$ This completes the proof.

**Theorem 2** Consider a CFG T=(σ,μ) with |σ*| = *n*. Then

$$\begin{array}{c} \begin{array}{c} 0\leq \left\langle \left[{CI}^{-}\left(T\right),{CI}^{+}\left(T\right)\right],{CI}^{F}\left(T\right)\right\rangle \leq \left\langle \left[{CI}^{-}\left(T^{\prime }\right),{CI}^{+}\left(T^{\prime }\right)\right],{CI}^{F}\left(T^{\prime }\right)\right\rangle , \end{array} \end{array}$$

where the vertex set of T spans $T^{\prime }=\left(\sigma^{\prime },\mu^{\prime }\right)$ and $T^{\prime }=\left(\sigma^{\prime },\mu^{\prime }\right)$ is complete CFG.

**Proof** Suppose T=(σ,μ) be a CFG. If |σ*| = 0, then $\langle \left[{CI}^{-}\left(T\right),{CI}^{+}\left(T\right)\right],{CI}^{F}\left(T\right)\rangle =0$. Take a complete CFG $T^{\prime }=\left(\sigma^{\prime },\mu^{\prime }\right)$ and order of σ* be *n* with $\langle \left[\sigma^{\prime -}\left(i\right),\sigma^{\prime +}\left(i\right)\right],\sigma^{\prime F}\left(i\right)\rangle =\langle \left[\sigma^{-}\left(i\right),\sigma^{+}\left(i\right)\right],\sigma^{F}\left(i\right)\rangle$. Then

$$\begin{array}{c} \left\langle \left[\mu^{-}\left(ij\right),\mu^{+}\left(ij\right)\right],\mu^{F}\left(ij\right)\right\rangle \leq \left\langle \left[\mu^{\prime -}\left(ij\right),\mu^{\prime +}\left(ij\right)\right],\mu^{\prime F}\left(ij\right)\right\rangle . \end{array}$$ (1)

This implies that,

$$\begin{array}{c} \begin{array}{c} \left\langle \left[{CONN}_{T}^{-}\left(i,j\right),{CONN}_{T}^{+}\left(i,j\right)\right],{CONN}_{T}^{F}\left(i,j\right)\right\rangle \leq \langle \left[{CONN}_{T^{\prime }}^{-}\left(i,j\right),{CONN}_{T^{\prime }}^{+}\left(i,j\right)\right],\\ {CONN}_{T^{\prime }}^{F}\left(i,j\right)\rangle , \end{array} \end{array}$$

for all *i*, *j* ∈ σ*. This further shows that

$$\begin{array}{c} \begin{array}{c} 0\leq \left\langle \left[{CI}^{-}\left(T\right),{CI}^{+}\left(T\right)\right],{CI}^{F}\left(T\right)\right\rangle \leq \left\langle \left[{CI}^{-}\left(T^{\prime }\right),{CI}^{+}\left(T^{\prime }\right)\right],{CI}^{F}\left(T^{\prime }\right)\right\rangle . \end{array} \end{array}$$

The subgraphs of a CFG obtained by vertex deletion or edge deletion reduce the values of many parameters related to connectivity. But in case of ${CI}^{\infty }$, these subgraphs depended on the type of the vertex and edge which is deleted from the graph.

**Proposition 1** Consider a CFG T=(σ,μ) and edge *ij* ∈ μ*, then $\langle \left[{CI}^{-}\left(T-ij\right),{CI}^{+}\left(T-ij\right)\right],{CI}^{F}\left(T-ij\right)\rangle \leq \langle \left[{CI}^{-}\left(T\right),{CI}^{+}\left(T\right)\right],{CI}^{F}\left(T\right)\rangle$.

**Theorem 3** Consider a CFG T=(σ,μ) and *ij* ∈ μ*. Then

$$\begin{array}{c} \left\langle \left[{CI}^{-}\left(T-ij\right),{CI}^{+}\left(T-ij\right)\right],{CI}^{F}\left(T-ij\right)\right\rangle <\left\langle \left[{CI}^{-}\left(T\right),{CI}^{+}\left(T\right)\right],{CI}^{F}\left(T\right)\right\rangle \end{array}$$

iff *ij* is *α*− strong.

**Proof** Assume that *ij* is *α*− strong. Then by definition,

$$\begin{array}{c} {CONN}_{T-ij}^{-}\left(i,j\right)<\mu^{-}\left(ij\right),{CONN}_{T-ij}^{+}\left(i,j\right)<\mu^{+}\left(ij\right),{CONN}_{T-ij}^{F}\left(i,j\right)<\mu^{F}\left(ij\right). \end{array}$$

This implies that

$$\begin{array}{c} \begin{array}{c} \left\langle \left[{CI}^{-}\left(T-ij\right),{CI}^{+}\left(T-ij\right)\right],{CI}^{F}\left(T-ij\right)\right\rangle <\left\langle \left[{CI}^{-}\left(T\right),{CI}^{+}\left(T\right)\right],{CI}^{F}\left(T\right)\right\rangle . \end{array} \end{array}$$

For converse, assume that

$$\begin{array}{c} \begin{array}{c} \left\langle \left[{CI}^{-}\left(T-ij\right),{CI}^{+}\left(T-ij\right)\right],{CI}^{F}\left(T-ij\right)\right\rangle <\left\langle \left[{CI}^{-}\left(T\right),{CI}^{+}\left(T\right)\right],{CI}^{F}\left(T\right)\right\rangle . \end{array} \end{array}$$ (2)

suppose on contrary, *ij* is not *α*− strong, then either ${CONN}_{T-ij}^{-}\left(i,j\right)\geq \mu^{-}\left(ij\right)$ or ${CONN}_{T-ij}^{+}\left(i,j\right)\geq \mu^{+}\left(ij\right)$ or ${CONN}_{T-ij}^{F}\left(i,j\right)\geq \mu^{F}\left(ij\right).$

This further shows that either ${CONN}_{T-ij}^{-}\left(i,j\right)={CONN}_{T}^{-}\left(i,j\right)$ or ${CONN}_{T-ij}^{+}\left(i,j\right)={CONN}_{T}^{+}\left(i,j\right)$ or ${CONN}_{T-ij}^{F}\left(i,j\right)={CONN}_{T}^{F}\left(i,j\right)$, respectively. In any case, this contradicts (2).

**Corollary 1** Let T=(σ,μ) be a CFG. Then $\langle \left[{CI}^{-}\left(T-ij\right),{CI}^{+}\left(T-ij\right)\right],{CI}^{F}\left(T-ij\right)\rangle =\langle \left[{CI}^{-}\left(T\right),{CI}^{+}\left(T\right)\right],{CI}^{F}\left(T\right)\rangle$ iff *ij* satisfies one of the followings:

- *ij* is *β*-strong.
- *ij* is partially *δ*- weak.

**Theorem 4** Let T=(σ,μ) be a CFG on *n* vertices and *m* edges such that $T^{⋆}$ is a tree. Let $d=\langle \left[d^{-},d^{+}\right],d^{F}\rangle =\langle \left[min_{a\in \sigma^{*}}\sigma^{-}\left(v\right),min_{a\in \sigma^{*}}\sigma^{+}\left(v\right)\right],min_{a\in \sigma^{*}}\sigma^{F}\left(v\right)\rangle$ and $p=\langle \left[p^{-},p^{+}\right],p^{F}\rangle$ = 〈[*max*_*a* ∈ σ*_σ^−^(*v*), *max*_*a* ∈ σ*_σ^+^(*v*)], $max_{a\in \sigma^{*}}\sigma^{F}\left(v\right)\rangle$. Then

$$d^{2}\left(S\left(T\right)+m_{1}d\right)\leq {CI}^{\infty }\left(T\right)\leq p^{2}\left(S\left(T\right)+m_{1}p\right),$$

where $m_{1}=\frac{n(n-1)}{2}-m.$

**Proof** By definition, we have

$$\begin{array}{c} \begin{array}{ccc} {CI}^{-}\left(T\right) & = & \sum_{v_{i},v_{j}\in \sigma^{*}}\sigma^{-}\left(v_{i}\right)\sigma^{-}\left(v_{j}\right){CONN}_{T}^{-}\left(v_{i},v_{j}\right)\\ & = & \sum_{v_{i}v_{j}\in \mu^{*}}\sigma^{-}\left(v_{i}\right)\sigma^{-}\left(v_{j}\right){CONN}_{T}^{-}\left(v_{i},v_{j}\right)+\\ & & \sum_{v_{i},v_{j}\notin \mu^{*}}\sigma^{-}\left(v_{i}\right)\sigma^{-}\left(v_{j}\right){CONN}_{T}^{-}\left(v_{i},v_{j}\right). \end{array} \end{array}$$

It is easy to see that for *uv* ∈ *μ*, ${CONN}^{\infty }\left(u,v\right)=\mu \left(uy\right)$ and for *uv* ∉ *μ*, ${CONN}^{\infty }\left(u,v\right)\geq d$. Therefore we have

$$\begin{array}{c} \begin{array}{ccc} \sum_{v_{i}v_{j}\in \mu^{*}}{\left(d^{-}\right)}^{2}\left(\mu^{-}\left(uv\right)\right)+\sum_{v_{i},v_{j}\notin \mu^{*}}{\left(d^{-}\right)}^{3}\leq {CI}^{-}\left(T\right) & \leq & \sum_{v_{i}v_{j}\in \mu^{*}}{\left(p^{-}\right)}^{2}\left(\mu^{-}\left(uv\right)\right)+\\ \sum_{v_{i},v_{j}\notin \mu^{*}}{\left(p^{-}\right)}^{3}\\ {\left(d^{-}\right)}^{2}\sum_{v_{i}v_{j}\in \mu^{*}}\left(\mu^{-}\left(uv\right)\right)+{\left(d^{-}\right)}^{3}\sum_{v_{i},v_{j}\notin \mu^{*}}1\leq {CI}^{-}\left(T\right) & \leq & \\ {\left(p^{-}\right)}^{2}\sum_{v_{i}v_{j}\in \mu^{*}}\left(\mu^{-}\left(uv\right)\right)+{\left(p^{-}\right)}^{3}\sum_{v_{i},v_{j}\notin \mu^{*}}1. \end{array} \end{array}$$

As *d*^−^ ≤ *σ*^+^(*i*) ≤ *p*^−^ for all *i* ∈ σ* and $\sum_{v_{i}v_{j}\in \mu^{*}}\left(\mu^{-}\left(uv\right)\right)=S^{-}\left(T\right)$, therefore, we can write

$$\begin{array}{c} \begin{array}{c} {\left(d^{-}\right)}^{2}S^{-}\left(T\right)+{\left(d^{-}\right)}^{3}\left(\frac{n(n-1)}{2}-m\right)\leq {CI}^{-}\left(T\right)\leq {\left(p^{-}\right)}^{2}S^{+}\left(T\right)+{\left(p^{-}\right)}^{3}\left(\frac{n(n-1)}{2}-m\right) \end{array} \end{array}$$

$$\begin{array}{c} \begin{array}{c} {\left(d^{-}\right)}^{2}\left(S^{-}\left(T\right)+d^{-}\left(\frac{n(n-1)}{2}-m\right)\right)\leq {CI}^{-}\left(T\right)\leq {\left(p^{-}\right)}^{2}\left(S^{-}\left(T\right)+p^{-}\left(\frac{n(n-1)}{2}-m\right)\right). \end{array} \end{array}$$ (3)

Similarly,

$$\begin{array}{c} \begin{array}{c} {\left(d^{+}\right)}^{2}\left(S^{+}\left(T\right)+d^{+}\left(\frac{n(n-1)}{2}-m\right)\right)\leq {CI}^{+}\left(T\right)\leq {\left(p^{+}\right)}^{2}\left(S^{+}\left(T\right)+p^{+}\left(\frac{n(n-1)}{2}-m\right)\right). \end{array} \end{array}$$ (4)

and

$$\begin{array}{c} \begin{array}{c} {\left(d^{F}\right)}^{2}\left(S^{F}\left(T\right)+d^{F}\left(\frac{n(n-1)}{2}-m\right)\right)\leq {CI}^{F}\left(T\right)leq{\left(p^{F}\right)}^{2}\left(S^{F}\left(T\right)+p^{F}\left(\frac{n(n-1)}{2}-m\right)\right). \end{array} \end{array}$$ (5)

Let $m_{1}=\frac{n(n-1)}{2}-m$, then from Eqs (3), (4) and (5), we have

$$\begin{array}{c} \begin{array}{c} \left\langle \left[{\left(d^{-}\right)}^{2}\left(S^{-}\left(T\right)+{\left(d^{-}\right)}^{3}m_{1}\right),{\left(d^{+}\right)}^{2}\left(S^{+}\left(T\right)+d^{+}m_{1}\right)\right],{\left(d^{F}\right)}^{2}\left(S^{F}\left(T\right)+d^{F}m_{1}\right)\right\rangle \end{array} \end{array}$$

$$\begin{array}{c} \begin{array}{c} \leq {CI}^{\infty }\left(T\right)\leq \left\langle \left[{\left(p^{-}\right)}^{2}\left(S^{-}\left(T\right)+{\left(p^{-}\right)}^{3}m_{1}\right),{\left(p^{+}\right)}^{2}\left(S^{+}\left(T\right)+d^{-}m_{1}\right)\right],{\left(p^{F}\right)}^{2}\left(S^{F}\left(T\right)+p^{F}m_{1}\right)\right\rangle \end{array} \end{array}$$

$$\begin{array}{c} \begin{array}{c} \left\langle \left[{\left(d^{-}\right)}^{2},{\left(d^{+}\right)}^{2}\right],{\left(d^{F}\right)}^{2}\right\rangle \left(\left\langle \left[S^{-}\left(T\right),S^{+}\left(T\right)\right],S^{F}\left(T\right)\right\rangle +m_{1}\left\langle \left[d^{-},d^{+}\right],d^{F}\right\rangle \right) \end{array} \end{array}$$

$$\begin{array}{c} \begin{array}{c} \leq {CI}^{\infty }\left(T\right)\leq {\left\langle \left[{\left(p^{-}\right)}^{2},{\left(p^{+}\right)}^{2}\right],p^{F}\right)}^{2}\rangle (\left\langle \left[S^{-}\left(T\right),S^{+}\left(T\right)\right],S^{F}\left(T\right)\right\rangle +m_{1}\left\langle \left[p^{-},p^{+}\right],\left(p^{F}\right\rangle \right) \end{array} \end{array}$$ (6)

Hence, we get $d^{2}\left(S\left(T\right)+m_{1}d\right)\leq {CI}^{\infty }\left(T\right)\leq p^{2}\left(S\left(T\right)+m_{1}p\right)$.

**Definition 11** A CFG graph T is referred as CF cycle if its crisp graph $T^{⋆}$ is a cycle and T contains no partial *δ*− weak edge.

**Theorem 5** Let T=(σ,μ) be a partial saturated CFC with *n* vertices and *m* edges such that every partial *α*− strong edge is equal to $\langle \left[a^{-},a^{+}\right],a^{F}\rangle$ and every *β*− edge is equal to $\langle \left[b^{-},b^{+}\right],b^{F}\rangle$. Moreover $\langle \left[\sigma^{-}\left(x\right),\sigma^{+}\left(x\right)\right],\sigma^{F}\left(x\right)\rangle =\langle \left[c^{-},c^{+}\right],c^{F}\rangle =c$. Then

$${CI}^{\infty }\left(T\right)=\frac{nc}{2}\left(a+\left(n-2\right)b\right).$$

**Proof** Since T is partial saturated, every vertex must be incident with one or more partial *α*− strong and one or more *β*− strong edges. As each vertex is incident with exactly two vertices, so each partial *α*− strong edge is adjacent to a *β*− edge. This is only possible if *n* is even. Now by definition, we have

$$\begin{array}{ccc} {CI}^{-}\left(T\right) & = & \sum_{v_{i},v_{j}\in \sigma^{*}}\sigma^{-}\left(v_{i}\right)\sigma^{-}\left(v_{j}\right){CONN}_{T}^{-}\left(v_{i},v_{j}\right) \end{array}$$ (7)

$$\begin{array}{ccc} & = & \sum_{v_{i}v_{j}\in \mu^{*}}\sigma^{-}\left(v_{i}\right)\sigma^{-}\left(v_{j}\right){CONN}_{T}^{-}\left(v_{i},v_{j}\right) \end{array}$$ (8)

$$\begin{array}{ccc} & + & \sum_{v_{i},v_{j}\notin \mu^{*}}\sigma^{-}\left(v_{i}\right)\sigma^{-}\left(v_{j}\right){CONN}_{T}^{-}\left(v_{i},v_{j}\right). \end{array}$$ (9)

It is easy to see that for *uv* ∈ μ*, ${CONN}^{\infty }\left(u,v\right)=\mu \left(uy\right)$. Now for *uv* ∉ *μ*, each path must contain one or more partial *α*− strong and one or more *β*− strong edges. Thus ${CONN}^{\infty }\left(u,v\right)=\langle \left[a^{-},a^{+}\right],a^{F}\rangle \wedge \langle \left[b^{-},b^{+}\right],b^{F}\rangle =\langle \left[b^{-},b^{+}\right],b^{F}\rangle$. Therefore Eq (8) becomes

$$\begin{array}{c} {CI}^{-}\left(T\right)=\sum_{v_{i}v_{j}\in \mu^{*}}{\left(c^{-}\right)}^{2}\mu^{-}\left(v_{i}v_{j}\right)+\sum_{v_{i},v_{j}\notin \mu^{*}}{\left(c^{-}\right)}^{2}b^{-}. \end{array}$$ (10)

Now *μ*^−^(*v*_*i*_*v*_*j*_) = *a*^−^ or *μ*^−^(*v*_*i*_*v*_*j*_) = *b*^−^ and as *n* is even so half of the edges have membership *a*^−^ and other have membership *b*^−^. Thus from (8), we get

$$\begin{array}{ccc} {CI}^{-}\left(T\right) & = & \frac{n}{2}{\left(c^{-}\right)}^{2}\left(a^{-}+b^{-}\right)+{\left(c^{-}\right)}^{2}b^{-}\left(\frac{n(n-1)}{2}-n\right)\\ & = & \frac{n}{2}{\left(c^{-}\right)}^{2}\left(a^{-}+b^{-}\right)+{\left(c^{-}\right)}^{2}b^{-}\left(\frac{n(n-3)}{2}\right)\\ & = & \frac{{\left(c^{-}\right)}^{2}n}{2}\left(a^{-}+b^{-}+b^{-}\left(n-3\right)\right)\\ & = & \frac{{\left(c^{-}\right)}^{2}n}{2}\left(a^{-}+b^{-}\left(n-2\right)\right). \end{array}$$ (11)

Similarly, we can write

$$\begin{array}{c} {CI}^{+}\left(T\right)=\frac{{\left(c^{+}\right)}^{2}n}{2}(a^{+}+b^{+}\left(n-2\right). \end{array}$$ (12)

and

$$\begin{array}{c} {CI}^{F}\left(T\right)=\frac{{\left(c^{F}\right)}^{2}n}{2}(a^{F}+b^{F}\left(n-2\right). \end{array}$$ (13)

Combining Eqs (11), (12) and (13), we get

$$\begin{array}{c} \begin{array}{c} {CI}^{\infty }\left(T\right)=\frac{n}{2}\left\langle \left[{\left(c^{-}\right)}^{2},{\left(c^{+}\right)}^{2}\right],{\left(c^{F}\right)}^{2}\right\rangle \left(\left\langle \left[{\left(a^{-}\right)}^{2},{\left(a^{+}\right)}^{2}\right],{\left(a^{F}\right)}^{2}\right\rangle \right)\\ +\left(n-2\right)\left\langle \left[{\left(b^{-}\right)}^{2},{\left(b^{+}\right)}^{2}\right],{\left(b^{F}\right)}^{2}\right\rangle =\frac{nc}{2}\left(a+\left(n-2\right)b\right). \end{array} \end{array}$$

This completes the proof.

## 5 Average connectivity index of a cubic fuzzy graph

**Definition 12** Average connectivity index $\left({ACI}^{\infty }\right)$ of T=(σ,μ) is denoted by ${ACI}^{\infty }\left(T\right)$ and defined as

$$\begin{array}{ccc} {ACI}^{\infty }\left(T\right) & = & \langle \left[{ACI}^{-}\left(T\right),{ACI}^{+}\left(T\right)\right],{ACI}^{F}\left(T\right)\rangle , \end{array}$$

where

ACI+(T)=1(n2)∑(i,j)∈σ*σ+(i)σ+(j)CONNT+(i,j),ACI-(T)=1(n2)∑(i,j)∈σ*σ-(i)σ-(j)CONNT-(i,j),ACIF(T)=1(n2)∑(j,j)∈σ*σF(i)σF(j)CONNTF(i,j).

**Definition 13** A PCCRN (partial cubic connectivity reducing node) of a CFG T=(σ,μ) is a node *i* ∈ σ* if one of the following hold:

1.
$\left[{ACI}^{-}\left(T\right),{ACI}^{+}\left(T\right)\right]>\left[{ACI}^{-}\left(T-i\right),{ACI}^{+}\left(T-i\right)\right]$ and ${ACI}^{F}\left(T\right)\geq {ACI}^{F}\left(T-i\right)$
2.
$\left[{ACI}^{-}\left(T\right),{ACI}^{+}\left(T\right)\right]\geq \left[{ACI}^{-}\left(T-i\right),{ACI}^{+}\left(T-i\right)\right]$ and ${ACI}^{F}\left(T\right)>{ACI}^{F}\left(T-i\right)$

If (1) holds, then *i* is referred as *IVF*− connectivity reducing node, whereas if (2) is satisfied, then it is referred as *F*− connectivity reducing node. If both (1) and (2) are satisfied, then it is referred as connectivity reducing node.

**Definition 14** A PCCEN (partial cubic connectivity enhancing node) of a CFG T=(σ,μ) is a node *i* ∈ σ* if one of the following hold

1.
$\left[{ACI}^{-}\left(T\right),{ACI}^{+}\left(T\right)\right]<\left[{ACI}^{-}\left(T-i\right),{ACI}^{+}\left(T-i\right)\right]$ and ${ACI}^{F}\left(T\right)\leq {ACI}^{F}\left(T-i\right)$
2.
$\left[{ACI}^{-}\left(T\right),{ACI}^{+}\left(T\right)\right]\leq \left[{ACI}^{-}\left(T-i\right),{ACI}^{+}\left(T-i\right)\right]$ and ${ACI}^{F}\left(T\right)<{ACI}^{F}\left(T-i\right)$

If (1) holds, then *i* is referred as *IVF*− connectivity enhancing node, whereas if (2) is satisfied, then it is referred as *F*− connectivity enhancing node. If both (1) and (2) are satisfied, then it is referred as connectivity enhancing node.

**Definition 15** A neutral node of a CFG T=(σ,μ) is a node *i* ∈ σ* if it satisfies:

${ACI}^{-}\left(T-i\right)={ACI}^{-}\left(T\right),{ACI}^{+}\left(T-i\right)={ACI}^{+}\left(T\right)$ and ${ACI}^{F}\left(T-i\right)={ACI}^{F}\left(T\right)$.

**Definition 16** A PCCEG (partial cubic connectivity enhancing graph) is CFG T=(σ,μ) if there are one or more PCCENs in T=(σ,μ). Whereas, A PCCRG (partial cubic connectivity reducing graph) is CFG T=(σ,μ) if it has no PCCENs and there are one or more PCCRNs in T=(σ,μ). If all the vertices of T are neutral, then it is referred as neutral graph.

**Proposition 2** Let T=(σ,μ) be a CFG and *i* ∈ σ* with *n* = |σ*| ≥ 3. Let $\langle \left[r^{-},r^{+}\right],r^{F}\rangle =r=\langle \left[\frac{{CI}^{-}\left(T\right)}{{CI}^{-}\left(T-i\right)},\frac{{CI}^{+}\left(T\right)}{{CI}^{+}\left(T-i\right)}\right],\frac{{CI}^{F}\left(T\right)}{{CI}^{F}\left(T-i\right)}\rangle$, then *i* is PCCEN if and only if $\left[r^{-},r^{+}\right]<\frac{n}{n-2}\left[1,1\right]$ and $r^{F}\leq \frac{n}{n-2}$ or $\left[r^{-},r^{+}\right]\leq \frac{n}{n-2}\left[1,1\right]$ and $r^{F}<\frac{n}{n-2}$. The vertex *i* is PCCRN if and only if $\left[r^{-},r^{+}\right]>\frac{n}{n-2}\left[1,1\right]$ and $r^{F}\geq \frac{n}{n-2}$ or $\left[r^{-},r^{+}\right]\geq \frac{n}{n-2}\left[1,1\right]$ and $r^{F}>\frac{n}{n-2}$ and *i* is neutral if and only if $\langle \left[r^{-},r^{+}\right],r^{F}\rangle =r=\frac{n}{n-2}\langle \left[1,1\right],1\rangle$.

**Proof** By definition the node *i* is a neutral node if and only if ${ACI}^{-}\left(T\right)={ACI}^{-}\left(T-i\right),{ACI}^{+}\left(T\right)={ACI}^{+}\left(T-i\right),$ and ${ACI}^{F}\left(T\right)={ACI}^{F}\left(T-i\right).$ Now again by definition, we know that ACI-(T)=1(n2)(CI-(T)), ACI+(T)=1(n2)(CI+(T)), ACIF(T)=1(n2)(CIF(T)) and ACI-(T-i)=1(n-12)(CI-(T-i)), ACI+(T-i)=1(n-12)(CI+(T-i)), ACIF(T-i)=1(n-12)(CIF(T-i)). Therefore, for ${ACI}^{-}\left(T\right)$, we have

ACI-(T)=ACI-(T-i)1(n2)(CI-(T))=1(n-12)(CI-(T-i))CI-(T)CI-(T-i)=(n2)(n-12)CI-(T)CI-(T-i)=nn-2

Thus $r^{-}=\frac{n}{n-2}$ Similarly

r+=CI+(T)CI+(T-i)=(n2)(n-12),

and

$$\begin{array}{ccc} r^{F}=\frac{{CI}^{F}\left(T\right)}{{CI}^{F}\left(T-i\right)} & = & \frac{n}{n-2}. \end{array}$$

Hence *i* is neutral node if and only if

$$\begin{array}{ccc} r & = & \left\langle \left[r^{-},r^{+}\right],r^{F}\right\rangle \\ & = & \left\langle \left[\frac{{CI}^{-}\left(T\right)}{{CI}^{-}\left(T-i\right)},\frac{{CI}^{+}\left(T\right)}{{CI}^{+}\left(T-i\right)}\right],\frac{{CI}^{F}\left(T\right)}{{CI}^{F}\left(T-i\right)}\right\rangle \\ & = & \left\langle \left[\frac{n}{n-2},\frac{n}{n-2}\right],\frac{n}{n-2}\right\rangle \\ & = & \frac{n}{n-2}\left\langle \left[1,1\right],1\right\rangle \end{array}$$

The proofs for PCCRN and PCCEN are similar.

## 6 Application to determine danger zone of tsunami threat

Natural disasters are events that are caused by natural phenomena and can have devastating consequences for the environment, human populations and infrastructure. They can take many different forms, including floods, hurricanes, earthquakes, tsunamis, tornadoes, wildfires and volcanic eruptions. One of the defining characteristics of natural disasters is their unpredictability. When natural disasters strike, they can cause widespread destruction and loss of life. They can also disrupt entire economies, causing significant financial losses and exacerbating social and political tensions.

Earthquakes are one of the most destructive and unpredictable natural disasters. An earthquake is a sudden rapid shaking of the ground caused by the movement of tectonic plates. It can cause significant damage to buildings and infrastructure, as well as trigger secondary hazards such as tsunamis, landslides and fires. The impact of earthquakes can widespread damage to buildings, roads, bridges and ports, as well as disruptions to essential services such as electricity and water. Secondary hazards such as tsunamis, landslides and fires can also exacerbate the impact of the disaster.

Earthquakes are a major natural hazard that can have a significant impact on communities and economies. To reduce the impact of earthquakes, it is important to invest in disaster risk reduction measures and emergency response planning, as well as to build infrastructure that is able to withstand earthquakes and other natural hazards.

Therefore, here we discuss the impact of earthquakes in certain areas by using cubic *α*−strong edges, cubic *β*−strong edges, cubic *δ*−weak edges, partial cubic *α*−strong edges and partial cubic *δ*−weak edges.

For this purpose, consider the problem in which an earthquake take place in deep ocean. A team from Pacific Tsunami Warning Center (PTWC) has to decide to find the region which is in danger zone of tsunami threat.

### 6.1 Tsunami threat model

With the help of CFG, a tsunami threat model is developed. In this tsunami threat model, vertices correspond to different areas with lower *IVF*-membership values indicating past tsunami threat values, upper *IVF*-membership values indicating future tsunami threat values and *F*-membership values indicating current tsunami threat values. The edges in this system represent the possibility of a danger zone arising due to a tsunami threat. By analyzing the strength of the connectedness between different areas, we can classify the types of danger zones into five categories: cubic *α*−strong zone, cubic *β*−strong zone, cubic *δ*−weak zone, partial cubic *α*−strong zone and partial cubic *δ*−weak zone. A cubic *α*−strong zone represents area with no tsunami threat, a partial cubic *α*−strong zone represents area with a very low tsunami threat, a cubic *β*−strong zone represents area with a low tsunami threat, a partial cubic *δ*−weak zone represents areas with a high tsunami threat and a cubic *δ*−weak zone represents area with a very high tsunami threat.

**Fig 3:**
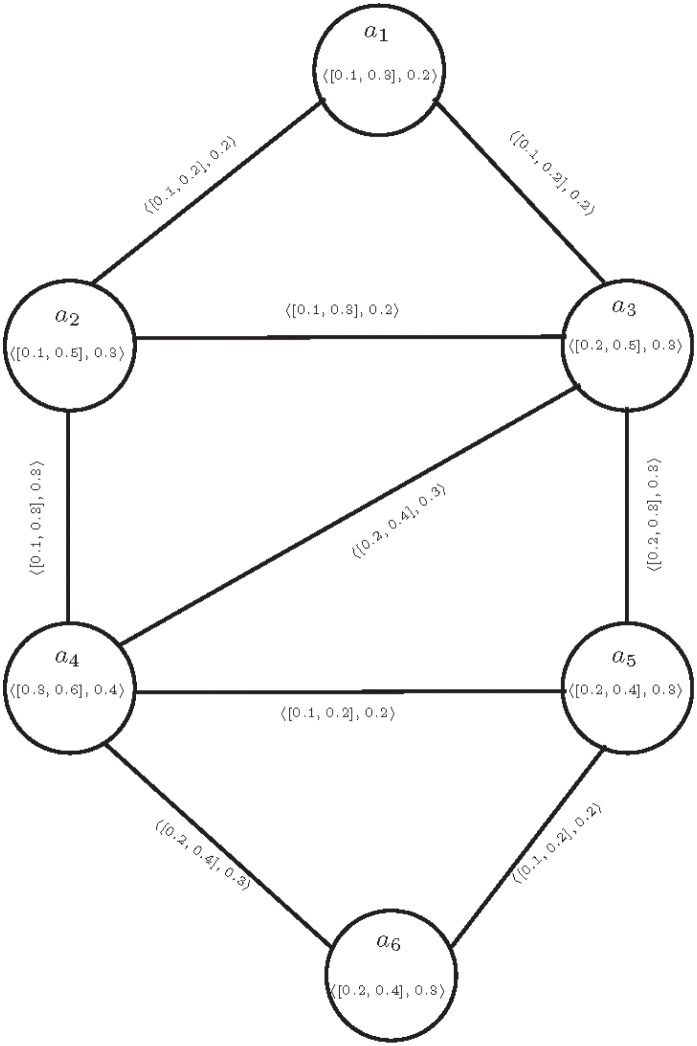
T=(I,J) .

FGs are two-dimensional models that represent the relationships between elements and their degree of membership using nodes and edges. Dealing with ambiguous data and discussing the nature of each edge in a FG can be complex when utilizing fuzzy theory. On the other hand, CFGs are an improved approach to FGs. The membership values of vertices and edges in a fuzzy graph are between 0 and 1, whereas CFGs are more significant because vertices and edges have both lower and upper IVF-membership values and F-membership values. These membership values can be any real number in the interval [0, 1]. CFGs are an effective approach to deal with inadequate information of relationships among areas and controlling information loss within a given system. An algorithm to identify the affecting areas due to tsunami threat is shown in Table 2. Consider the set *X* consisting of the areas *a*_1_, *a*_2_, *a*_3_, *a*_4_, *a*_5_ and *a*_6_, in the vicinity of an ocean where an earthquake takes place and these areas can be affected by tsunami. A tsunami threat model is developed with the help of CFG T=(I,J) shown in Fig 3. The IVF-memberships and F-memberships of the vertices of T=(I,J) and edges of T=(I,J) are given in Tables 3 and 4, respectively.

**Table 2:** Algorithm.

**Algorithm: To Identify the affecting Area**
**Step 1**. Consider a cubic fuzzy tsunami network.
**Step 2**. Insert the areas set *X* = {*a*_1_, *a*_2_, …, *a*_*n*_} as the vertex set of CFG.
**Step 3**. Insert the value of membership of each edges in CFG.
**Step 4**. Calculate the strength of each pair of vertices by using formula,
$S\left(P\right)=\langle \left[L^{-}\left(P\right),L^{+}\left(P\right)\right],L^{F}\left(P\right)\rangle ,$
where
$L^{+}\left(P\right)=\wedge_{i=1}^{n}\mu^{+}\left(a_{i-1},a_{i}\right),$
$L^{-}\left(P\right)=\wedge_{i=1}^{n}\mu^{-}\left(a_{i-1},a_{i}\right),$
$L^{F}\left(P\right)=\wedge_{i=1}^{n}\mu^{F}\left(a_{i-1},w_{i}\right).$
**Step 5**. Calculate the strength of connectivity of each pair of vertices by
using formula
${CONN}_{T}^{\infty }\left(a_{i-1},a_{i}\right)=\langle \left[{CONN}_{T}^{-}\left(a_{i-1},a_{i}\right),{CONN}_{T}^{+}\left(a_{i-1},a_{i}\right)\right],{CONN}_{T}^{F}\left(a_{i-1},a_{i}\right)\rangle ,$
where
${CONN}_{T}^{+}\left(a_{i-1},a_{i}\right)=\vee_{P}\left\{L^{+}\left(P\right):P\;\text{is}\;\text{a}\;\text{path}\;\text{between}\;a_{i-1}\;\text{and}\;a_{i}\right\},$
${CONN}_{T}^{-}\left(a_{i-1},a_{i}\right)=\vee \left\{L^{-}\left(P\right):P\;\text{is}\;\text{a}\;\text{path}\;\text{between}\;a_{i-1}\;\text{and}\;a_{i}\right\},$
${CONN}_{T}^{F}\left(a_{i-1},a_{i}\right)=\vee \left\{L^{F}\left(P\right):P\;\text{is}\;\text{a}\;\text{path}\;\text{between}\;a_{i-1}\;\text{and}\;a_{i}\right\}.$
**Step 6**.(i): If $\mu^{+}\left(a_{i-1}a_{i}\right)>{CONN}_{T-a_{i-1}a_{i}}^{+}\left(a_{i-1},a_{i}\right),$
$\mu^{-}\left(a_{i-1}a_{i}\right)>{CONN}_{T-a_{i-1}a_{i}}^{-}\left(a_{i-1},a_{i}\right),$
$\mu^{F}\left(a_{i-1}a_{i}\right)>{CONN}_{T-a_{i-1}a_{i}}^{F}\left(a_{i-1},a_{i}\right),$
then the edge *a*_*i*−1_*a*_*i*_ is cubic *α*−strong zone
(ii): If
$\left[\mu_{T}^{-}\left(a_{i-1},a_{i}\right),\mu_{T-a_{i-1}a_{i}}^{+}\left(a_{i-1},a_{i}\right)\right]\geq \left[{CONN}_{T-a_{i-1}a_{i}}^{-}\left(a_{i-1},a_{i}\right),{CONN}_{T-a_{i-1}a_{i}}^{+}\left(a_{i-1},a_{i}\right)\right],$
$\mu_{T}^{F}\left(a_{i-1}a_{i}\right)>{CONN}_{T-a_{i-1}a_{i}}^{F}\left(a_{i-1},a_{i}\right)$
OR
If $\left[\mu_{T}^{-}\left(a_{i-1}a_{i}\right),\mu_{T}^{+}\left(a_{i-1}a_{i}\right)\right]>\left[{CONN}_{T-a_{i-1}a_{i}}^{-}\left(a_{i-1},a_{i}\right),{CONN}_{T-a_{i-1}a_{i}}^{+}\left(a_{i-1},a_{i}\right)\right],$
$\mu_{T}^{F}\left(a_{i-1}a_{i}\right)\geq {CONN}_{T-a_{i-1}a_{i}}^{F}\left(a_{i-1},a_{i}\right),$
then *a*_*i*−1_*a*_*i*_ is partial cubic *α*−strong zone
(iii): If $\mu^{+}\left(a_{i-1}a_{i}\right)={CONN}_{T-a_{i-1}a_{i}}^{+}\left(a_{i-1},a_{i}\right),$
$\mu^{-}\left(a_{i-1}a_{i}\right)={CONN}_{T-a_{i-1}a_{i}}^{-}\left(a_{i-1},a_{i}\right),$
$\mu^{F}\left(a_{i-1}a_{i}\right)={CONN}_{T-a_{i-1}a_{i}}^{F}\left(a_{i-1},a_{i}\right)$
then *a*_*i*−1_*a*_*i*_, then *a*_*i*−1_*a*_*i*_ is cubic *β*−strong zone.
(iv): If $\mu^{+}\left(a_{i-1}a_{i}\right)<{CONN}_{T-a_{i-1}a_{i}}^{+}\left(a_{i-1},a_{i}\right),$
$\mu^{-}\left(a_{i-1}a_{i}\right)<{CONN}_{T-a_{i-1}a_{i}}^{-}\left(a_{i-1},a_{i}\right),$
$\mu^{F}\left(a_{i-1}a_{i}\right)<{CONN}_{T-a_{i-1}a_{i}}^{F}\left(a_{i-1},a_{i}\right)$
then *a*_*i*−1_*a*_*i*_, then *a*_*i*−1_*a*_*i*_ is cubic *δ*−weak zone.
(v): If $\left[\mu_{T}^{-}\left(a_{i-1}a_{i}\right),\mu_{T}^{+}\left(a_{i-1}a_{i}\right)\right]\leq \left[{CONN}_{T-a_{i-1}a_{i}}^{-}\left(a_{i-1},a_{i}\right),{CONN}_{T-a_{i-1}a_{i}}^{+}\left(a_{i-1},a_{i}\right)\right],$
$\mu_{T}^{F}\left(a_{i-1}a_{i}\right)<{CONN}_{T-a_{i-1}a_{i}}^{F}\left(a_{i-1},a_{i}\right)$
OR
If $\left[\mu_{T}^{-}\left(a_{i-1}a_{i}\right),\mu_{T}^{+}\left(a_{i-1}a_{i}\right)\right]<\left[{CONN}_{T-a_{i-1}a_{i}}^{-}\left(a_{i-1},a_{i}\right),{CONN}_{T-a_{i-1}a_{i}}^{+}\left(a_{i-1},a_{i}\right)\right],$
and $\mu^{F}\left(a_{i-1}a_{i}\right)\leq {CONN}_{T-a_{i-1}a_{i}}^{F}\left(a_{i-1},a_{i}\right),$
then *a*_*i*−1_*a*_*i*_ is partial cubic *δ*−weak zone

**Table 3:** Membership value of each vertex in CFG T=(I,J).

Vertices	Vertex membership
*a*_1_	〈[0.1, 0.3], 0.2〉
*a*_2_	〈[0.1, 0.5], 0.3〉
*a*_3_	〈[0.2, 0.5], 0.3〉
*a*_4_	〈[0.3, 0.6], 0.4〉
*a*_5_	〈[0.2, 0.4], 0.3〉
*a*_6_	〈[0.2, 0.4], 0.3〉

**Table 4:** Membership value of each edge in CFG T=(I,J).

Edges	Edge membership	Edges	membership
(*a*_1_, *a*_2_)	〈[0.1, 0.2], 0.2〉	(*a*_3_, *a*_5_)	〈[0.2, 0.3], 0.3〉
(*a*_1_, *a*_3_)	〈[0.1, 0.2], 0.2〉	(*a*_4_, *a*_5_)	〈[0.1, 0.2], 0.2〉
(*a*_2_, *a*_3_)	〈[0.1, 0.3], 0.2〉	(*a*_4_, *a*_6_)	〈[0.2, 0.4], 0.3〉
(*a*_2_, *a*_4_)	〈[0.1, 0.3], 0.3〉	(*a*_5_, *a*_6_)	〈[0.1, 0.2], 0.2〉
(*a*_3_, *a*_4_)	〈[0.2, 0.4], 0.3〉		

List of all possible paths including the strengths and the strengths of their connections between *a*_1_ and *a*_2_ in a CFG are given in Table 5. Here, the edge (*a*_1_, *a*_2_) in CFG is cubic *α*−strong. Likewise, it would be worthwhile to investigate the nature of other edges between areas. Analyzing the characteristics of each edge in the CFG would further underscore the significance and efficacy of our research. Based on Fig 3 and conventional computations, the connectivity between vertices in T=(I,J) can be determined as follows:

$$\begin{array}{c} \begin{array}{ccc} & & {CONN}_{T-(a_{1}a_{2})}^{\infty }\left(a_{1},a_{2}\right)=\left\langle \left[0.1,0.2\right],0.2\right\rangle ,\\ & & {CONN}_{T-(a_{1}a_{3})}^{\infty }\left(a_{1},a_{3}\right)=\left\langle \left[0.1,0.2\right],0.2\right\rangle ,\\ & & {CONN}_{T-(a_{2}a_{3})}^{\infty }\left(a_{2},a_{3}\right)=\left\langle \left[0.1,0.3\right],0.3\right\rangle ,\\ & & {CONN}_{T-(a_{2}a_{4})}^{\infty }\left(a_{2},a_{4}\right)=\left\langle \left[0.1,0.3\right],0.2\right\rangle ,\\ & & {CONN}_{T-(a_{3}a_{4})}^{\infty }\left(a_{3},a_{4}\right)=\left\langle \left[0.1,0.3\right],0.2\right\rangle ,\\ & & {CONN}_{T-(a_{3}a_{5})}^{\infty }\left(a_{3},a_{5}\right)=\left\langle \left[0.1,0.2\right],0.2\right\rangle ,\\ & & {CONN}_{T-(a_{4}a_{5})}^{\infty }\left(a_{4},a_{5}\right)=\left\langle \left[0.2,0.3\right],0.3\right\rangle ,\\ & & {CONN}_{T-(a_{4}a_{6})}^{\infty }\left(a_{4},a_{6}\right)=\left\langle \left[0.1,0.2\right],0.2\right\rangle ,\\ & & {CONN}_{T-(a_{5}a_{6})}^{\infty }\left(a_{5},a_{6}\right)=\left\langle \left[0.2,0.3\right],0.3\right\rangle . \end{array} \end{array}$$ (14)

**Table 5:** All paths from *a*_1_ to *a*_2_ in T.

In CFG T
$P_{1}$ : *a*_1_ → *a*_2_ with strength 〈[0.1, 0.2], 0.2〉
$P_{2}$ : *a*_1_ → *a*_3_ → *a*_2_ with strength 〈[0.1, 0.2], 0.2〉
$P_{3}$ : *a*_1_ → *a*_3_ → *a*_4_ → *a*_2_ with strength 〈[0.1, 0.2], 0.2〉
$P_{4}$ : *a*_1_ → *a*_3_ → *a*_5_ → *a*_4_ → *a*_2_ with strength 〈[0.1, 0.2], 0.2〉
$P_{5}$ : *a*_1_ → *a*_3_ → *a*_5_ → *a*_6_ → *a*_4_ → *a*_2_ with strength 〈[0.1, 0.2], 0.2〉
${CONN}_{T}^{\infty }\left(a_{1},a_{2}\right)=\langle \left[0.1,0.2\right],0.2\rangle$
${CONN}_{T-(a_{1}a_{2})}^{\infty }\left(a_{1},a_{2}\right)=\langle \left[0.1,0.2\right],0.2\rangle$

It is noted that cubic *α*−strong zones are (*a*_3_, *a*_5_), (*a*_3_, *a*_4_), (*a*_4_, *a*_6_), cubic *β*−strong zones are (*a*_1_, *a*_2_), (*a*_1_, *a*_3_), cubic *δ*−weak zones are (*a*_4_, *a*_5_), (*a*_5_, *a*_6_), there is only one partial cubic *α*−strong zone which is (*a*_2_, *a*_4_) and partial cubic *δ*− weak zone is (*a*_2_, *a*_3_) in CFG system. The classification of areas with tsunami threat into different zones, will be helpful to interpret the situation of tsunami threat in areas due to earthquake. Based on the categorization of different zones according to the tsunami threat level the level of planning requires variation. In the cubic *α*−strong zone with no tsunami threat minimal planning is needed focusing on general disaster preparedness measures. A partial cubic *α*−strong zone requires moderate planning including early warning systems and resilient infrastructure. A cubic *β*−strong zone demands a higher level of planning with comprehensive emergency response plans and coastal protection measures. In a partial cubic *δ*−weak zone, extensive planning is necessary involving drills, evacuation centers and strict building codes. A cubic *δ*−weak zone representing a very high tsunami threat, requires the utmost level of planning including tsunami-resistant structures and advanced warning systems. Overall, planning efforts must align with the level of tsunami threat in each zone to ensure effective disaster risk reduction and mitigation measures. It is important to note that throughout this study, we specifically focused on simple connected CFGs. The concept of partial cubic *α*−strong and *δ*−weak edges is more advantageous compared to cubic strong and weak edges. This is because sometimes we encounter a problem or graph structure where the *IVF*-connectivity is either strictly less or greater than the *IVF*-membership value of an edge, while the *F*-connectivity equals to the *F*-membership value of that edge and vice versa. In such situations, the concept of cubic strong and weak edges fails to provide us with any relevant information about the nature of that edge, leading to difficulty in understanding it. In these conditions, the concept of partial cubic *α*−strong and *δ*−weak edges plays an important role by providing us with information about the nature of that edge. Hence, the concept of partial cubic *α*−strong and *δ*−weak edges is more beneficial compared to cubic strong and weak edges.

## 7 Comparative analysis

The concept of partial cubic *α*-strong and *δ*−weak edges presents a fresh expansion of the current notion of cubic *α*-strong and cubic *δ*-weak edges within the framework of earthquake-induced tsunami threat modeling. Through this comparative examination, it can be suggested that partial cubic *α*-strong and *δ*−weak edges provide specific advantages in contrast to cubic *α*-strong and cubic *δ*-weak edges.

$$\begin{array}{c} \begin{array}{ccc} & & {CONN}_{T-(a_{1}a_{2})}^{\infty }\left(a_{1},a_{2}\right)=\left\langle \left[0.1,0.2\right],0.2\right\rangle ,\\ & & {CONN}_{T-(a_{1}a_{3})}^{\infty }\left(a_{1},a_{3}\right)=\left\langle \left[0.1,0.2\right],0.2\right\rangle ,\\ & & {CONN}_{T-(a_{2}a_{3})}^{\infty }\left(a_{2},a_{3}\right)=\left\langle \left[0.1,0.3\right],0.3\right\rangle ,\\ & & {CONN}_{T-(a_{2}a_{4})}^{\infty }\left(a_{2},a_{4}\right)=\left\langle \left[0.1,0.3\right],0.2\right\rangle ,\\ & & {CONN}_{T-(a_{3}a_{4})}^{\infty }\left(a_{3},a_{4}\right)=\left\langle \left[0.1,0.3\right],0.2\right\rangle ,\\ & & {CONN}_{T-(a_{3}a_{5})}^{\infty }\left(a_{3},a_{5}\right)=\left\langle \left[0.1,0.2\right],0.2\right\rangle ,\\ & & {CONN}_{T-(a_{4}a_{5})}^{\infty }\left(a_{4},a_{5}\right)=\left\langle \left[0.2,0.3\right],0.3\right\rangle ,\\ & & {CONN}_{T-(a_{4}a_{6})}^{\infty }\left(a_{4},a_{6}\right)=\left\langle \left[0.1,0.2\right],0.2\right\rangle ,\\ & & {CONN}_{T-(a_{5}a_{6})}^{\infty }\left(a_{5},a_{6}\right)=\left\langle \left[0.2,0.3\right],0.3\right\rangle . \end{array} \end{array}$$ (15)

From Eq 15, when applying the concept of cubic *α*-strong, cubic *β*-strong, cubic *δ*-weak, partial cubic *α*-strong and partial cubic *δ*-weak edges to the tsunami threat model given in a Fig 3, specific edges can be identified with precision. These edges can be identified based on the satisfaction of the given edges conditions. Cubic *α*-strong edges are (*a*_3_, *a*_5_), (*a*_3_, *a*_4_), (*a*_4_, *a*_6_), cubic *β*-strong edges are (*a*_1_, *a*_2_), (*a*_1_, *a*_3_), cubic *δ*-weak edges are (*a*_4_, *a*_5_), (*a*_5_, *a*_6_), partial cubic *α*-strong edge is (*a*_2_, *a*_4_) and partial cubic *δ*− weak edge is (*a*_2_, *a*_3_). In Cubic Fuzzy Graph, when we discuss only cubic strong and weak edges, we have the concept of cubic *α*-strong edge. This edge is such that if we remove it and then check the strength of connectivity, the *IVF*-connectivity is strictly less than the *IVF*-membership value of that edge. In terms of *F*-connectivity, it’s also strictly less than the *F*-membership value. However, in the condition of a cubic *δ*-weak edge, it’s strictly greater. But in the case of a cubic *β*-strong edge, the *IVF*-connectivity is equal to the *IVF*-membership value, and the *F*-connectivity is equal to the *F*-membership value. At times, we encounter cases where the *IVF*-connectivity is equal to the *IVF*-membership value, but the *F*-connectivity is strictly less or greater than the *F*-membership value, or the *IVF*-connectivity is strictly less or greater than the *IVF*-membership value while the *F*-connectivity is equal to the *F*-membership value. In such situations, the concept of cubic *α*-strong or cubic *δ*-weak edges cannot handle this. Therefore, in such conditions, the concept of partial cubic *α*-strong and partial cubic *δ*-weak edges plays an important role. It provides information not only about cubic *α*-strong, cubic *β*-strong and cubic *δ*-weak edges but also about the nature of the remaining edges. From Fig 3 and Eq 15, if we only consider the concepts of cubic *α*-strong, cubic *β*-strong and cubic *δ*-weak edges, we have cubic *α*-strong zones as (*a*_3_, *a*_5_), (*a*_3_, *a*_4_), (*a*_4_, *a*_6_), cubic *β*-strong zones as (*a*_1_, *a*_2_), (*a*_1_, *a*_3_) and cubic *δ*-weak zones as (*a*_4_, *a*_5_), (*a*_5_, *a*_6_). However, the remaining edges that do not satisfy the conditions of these edges do not provide any information about their nature. Therefore, using the concepts of partial cubic *α*-strong and partial cubic *δ*-weak edges helps us understand the condition of the remaining edges. Hence, when viewed comparatively, the concept of partial cubic *α*-strong and *δ*-weak edges presents clear advantages over cubic *α*-strong and cubic *δ*-weak edges, particularly in terms of precise zone delineation and a thorough examination of tsunami conditions across past, present and future scenarios.

## 8 Conclusion

Fuzzy graphs play a crucial role in understanding and studying complex systems characterized by uncertain and imprecise information. From various kinds of fuzzy graphs, CFGs offer a more advantageous representation as compared to interval-valued fuzzy graphs and fuzzy graphs. This advantage stems from their ability to illustrate the membership degree of vertices and edges using both interval and fuzzy number forms. This enhanced representation facilitates a more profound and detailed understanding of the connections and uncertainties inherent in the graph’s structure. Connectivity or the strength of connectivity is always considered as a cornerstone in network theory. The connectivity in both fuzzy graphs and cubic fuzzy graphs involves comprehending cubic *α*−strong, cubic *β*−strong and cubic *δ*−weak edges. This comprehension is essential for analyzing intricate networks. Proficiency in these concepts significantly aids in decision-making, problem-solving and the analysis of various fields such as transportation, social networks and communication systems. The importance of connectivity and the comprehension of cubic fuzzy graphs have prompted a detailed discussion on connectivity within the domain of CFGs. In this research paper, the concepts of partial cubic *α*− strong and partial cubic *δ*− weak edges are introduced and bounds for the ${CI}^{\infty }$ of the CFG are computed. In scenarios, where we have information about the past, future and current conditions of a model or problem, we can represent the past condition as a lower interval-valued fuzzy membership, the future condition as an upper interval-valued fuzzy membership and the present condition as a fuzzy membership value. Our objective is to scrutinize the problem by deducing lower interval-valued fuzzy connectivity, upper interval-valued fuzzy connectivity and fuzzy connectivity. Furthermore, we aim to make new predictions based on this analysis. The average connectivity index ${ACI}^{\infty }$ depending upon the average strength of connectivity among vertices of a CFG is introduced. The concept of partial connectivity reducing node (PCRN) and partial connectivity enhancing node (PCEN) are introduced. To overcome the uncertainty in the economy and determine the impact of tsunami threat in different areas, an application by using strong and weak edges of CFG is proposed. Finally, a detailed comparison between our research results and the existing methods to showcase their applicability and productivity is provided. In the realm of future work, one promising avenue to explore is the hybridization of graph theory with recent advancements in Farmatean fuzzy set models given in [43–45]. This interdisciplinary approach has a lot of potential to advance both graph theory and Farmatean fuzzy set theory, paving the way for addressing intricate problems and improving decision-making procedures. We also want to extend the concept of vertex connectivity and edge connectivity to the cubic Intuitionistic fuzzy graph (CIFG).
